# Parliament and the Professions

**Published:** 1921-03-19

**Authors:** 


### Parliament and the Professions.

### Venereal Diseases Treatment Centres.

Dr. Addison, answering a question put by Sir J. D. Rees as to the effect of the suggestions of the National Birth-Rate Commission and the Society for the Prevention of Disease, stated that there is no intention of abolishing the 191 treatment centres which have been provided for the treatment of persons suffering from venereal diseases.

### Cancer.

Mr. Gilbert asked the Minister of Health whether his attention has been called to the recent increase of deaths from cancer, and whether his Department had made financial grants for treatment and research. Dr. Addison regretted the fact of the increase in deaths attributed to the disease, and called attention to the extensive work of the Imperial Cancer Research Fund, and of the action of the Medical Research Council in facilitating research into the possibilities of radio-therapy and the provision of substantial quantities of active radium salt for the purpose. He did not think the issues are sufficiently clear to recommend grants at the present time.

### Alien Health Precautions.

Sir H. Brittain questioned the Prime Minister as to the danger of disease being brought into the country by the aliens who cross for transhipment to Atlantic ports. The Home Secretary replied that these travellers are subject to certain regulations before their departure from the Continent, and the shipping companies which carry them are liable to heavy penalties in America for every diseased alien they take, and are obliged to re-ship such passengers. In addition, general precautions are taken in this country by the Ministry of Health and Port Sanitary Authorities.

### Malaria.

Dr. Addison informed Captain Elliott, in reference to steps taken to prevent the spread of malaria in England, that the subject was receiving close attention. The action of the Ministry was described in two recent Papers and in the Annual Report of the Chief Medical Officer for 1919-1920. The policy includes early treatment, protection of cases, and carriers against mosquito bites. Special supervision has been arranged in the Isle of Sheppey, where most of the infection in England has occurred. Locally contracted cases among the civil population have been reduced from 93 in 1919 to 32 in 1920.

### Maternity and Infant Welfare.

The Minister of Health stated that the death-rate of infants under one year of age per 1,000 births in recent years is as follows: 1914, 104.62; 1915, 109.72; 1916, 91.21; 1917, 96.5; 1918, 97.16; 1919, 89.13; 1920, 80. Grants amounting to £855,000 will be made during the current financial year towards the expenditure of local authorities on maternity and child welfare.

### Nation's Fund for Nurses.

A question was asked as to the Government's control over the Nation's Fund for Nurses, and in it Mr. J. Davison inquired whether the Lord Privy Seal was aware that, although one of the main objects was the benefit of present nurses who are in precarious circumstances owing to after-war conditions, or are suffering ill-health as a result of war strain, out of a total of nearly £150,000 only £2,144 had been applied to this purpose, whilst £40,000 had been given to the College of Nursing; and whether inquiries could be made into the administration of the Fund.

Mr. Lloyd George, who answered, said the Government had no control over the Fund, and suggested that a question should be addressed to the Charity Commissioners.

Mr. Lyle asked if it is not a fact that the suggestion made against the Fund has been made by ill-disposed people who were chagrined at not having started such a Fund themselves. (Hear, hear.) No answer was given.

